# Clinical Significance of *Escherichia albertii*

**DOI:** 10.3201/eid1803.111401

**Published:** 2012-03

**Authors:** Tadasuke Ooka, Kazuko Seto, Kimiko Kawano, Hideki Kobayashi, Yoshiki Etoh, Sachiko Ichihara, Akiko Kaneko, Junko Isobe, Keiji Yamaguchi, Kazumi Horikawa, Tânia A.T. Gomes, Annick Linden, Marjorie Bardiau, Jacques G. Mainil, Lothar Beutin, Yoshitoshi Ogura, Tetsuya Hayashi

**Affiliations:** University of Miyazaki, Miyazaki, Japan (T. Ooka, Y. Ogura, T. Hayashi);; Osaka Prefectural Institute of Public Health, Osaka, Japan (K. Seto);; Miyazaki Prefectural Institute for Public Health and Environment, Miyazaki (K. Kawano);; National Institute of Animal Health, Ibaraki, Japan (H. Kobayashi);; Fukuoka Institute of Health and Environmental Sciences, Fukuoka, Japan (Y. Etoh, S. Ichihara, K. Horikawa);; Yamagata Prefectural Institute of Public Health, Yamagata, Japan (A. Kaneko);; Toyama Institute of Health, Toyama, Japan (J. Isobe);; Hokkaido Institute of Public Health, Hokkaido, Japan (K. Yamaguchi);; Universidade Federal de São Paulo, São Paulo, Brazil (T.A.T. Gomes);; University of Liège, Liège, Belgium (A. Linden, M. Bardiau, J.G. Mainil);; Federal Institute for Risk Assessment, Berlin, Germany (L. Beutin)

**Keywords:** Escherichia albertii, attaching and effacing pathogen, intimin, enterohemorrhagic Escherichia coli, enteropathogenic Escherichia coli, Shiga toxin, locus of enterocyte effacement, bacteria

## Abstract

Discriminating *Escherichia albertii* from other *Enterobacteriaceae* is difficult. Systematic analyses showed that *E. albertii* represents a substantial portion of strains currently identified as *eae*-positive *Escherichia coli* and includes Shiga toxin 2f–producing strains. Because *E. albertii* possesses the *eae* gene, many strains might have been misidentified as enterohemorrhagic or enteropathogenic *E. coli.*

Attaching and effacing pathogens possess a locus of enterocyte effacement (LEE)–encoded type III secretion system. They form attaching and effacing lesions on intestinal epithelial cell surfaces by the combined actions of intimin, an *eae* gene–encoded outer membrane protein, and type III secretion system effectors. Attaching and effacing pathogens include enterohemorrhagic and enteropathogenic *Escherichia coli* (EHEC and EPEC, respectively) and *Citrobacter rodentium* (*1*,*2*). *Escherichia albertii* have recently been added to this group (*3*–*5*). However, the clinical significance of *E. albertii* has yet to be fully elucidated, partly because it is difficult to discriminate *E. albertii* from other *Enterobacteriaceae* spp. by using routine bacterial identification systems based on biochemical properties (*6*–*9*). A large number of *E. albertii* strains might have been misidentified as EPEC or EHEC because they possess the *eae* gene.

## The Study

**Figure 1:**
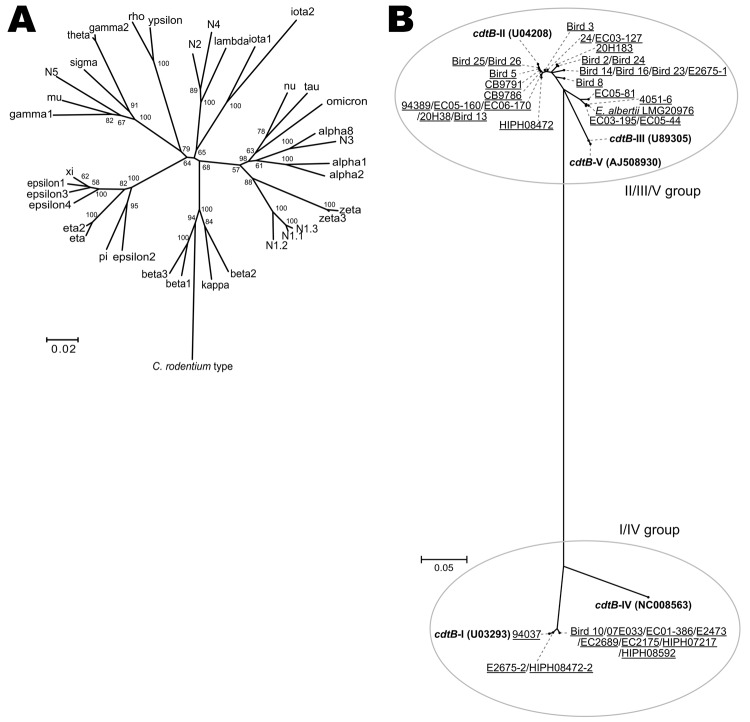
Phylogenies of the intimin subtypes and the *cdtB* genes of 275 *eae*-positive strains from humans, animals, and the environment that had been originally identified by routine diagnostic protocols as enteropathogenic or enterohemorrhagic *Escherichia coli*. A) Neighbor-joining tree constructed based on the amino acid sequences of 30 known intimin subtypes and previously undescribed 5 intimin subtypes (N1–N5) that were identified. The sequences of the N1–N5 alleles are substantially divergent from any of the known intimin subtypes (<95% sequence identity). Three variants of N1 (N1.1–N1.3) exhibit >95% homology to each other. B) Neighbor-joining tree constructed by using the partial amino acid sequences of the cytolethal distending toxin B subunit encoded by the *cdtB* gene. **Boldface** indicates reference sequences (and strain names) for 5 subtypes; underlining indicates alleles identified and names of the strains from which each allele was identified. The alleles that were amplified by the s2/as2 primer pair were classified into the I/IV subtype group, and those amplified by the s1/as1 primer pair were classified into the II/III/V subtype group (see Technical Appendix for primer information). Among the 3 alleles classified into the latter group, 1 was identified as a second copy in 2 *Escherichia albertii* strains (E2675–2 and HIPH08472–2), but the others were from either 1 *E. coli* strain (94037) or 8 *E. coli* strains (e.g., Bird 10). All alleles classified into the II/III/V subtype group were from *E. albertii* strains. Scale bars indicate amino acid substitutions (%) per site.

We collected 278 *eae*-positive strains that were originally identified by routine diagnostic protocols as EPEC or EHEC. They were isolated from humans, animals, and the environment in Japan, Belgium, Brazil, and Germany during 1993–2009 (Table 1; Technical Appendix). To characterize the strains, we first determined their intimin subtypes by sequencing the *eae* gene as described (Technical Appendix). Of the 275 strains examined, 267 possessed 1 of the 26 known intimin subtypes (4 subtypes—η, ν, τ, and a subtype unique to *C. rodentium*—were not found). In the remaining 8 strains, we identified 5 new subtypes; each showed <95% nt sequence identity to any known subtype, and they were tentatively named subtypes N1–N5. For subtype N1, 3 variants were identified (N1.1, N1.2, and N1.3, with >95% sequence identity among the 3 variants) (Figure 1, panel A).

**Table 1:** Summary of 275 *eae*-positive strains originally identified by routine diagnostic protocols as EPEC or EHEC

Origin	No. strains
Human, n = 193	
Symptomatic	154
Asymptomatic	7
No information	32
Animal, n = 76	
Bird	38
Pig	31
Cat	1
Deer	1
Bovid	1
Sheep	1
No information	3
Environment, n = 6	6

*EPEC, enteropathogenic *Escherichia coli*; EHEC, enterohemorrhagic *E. coli*.

**Figure 2:**
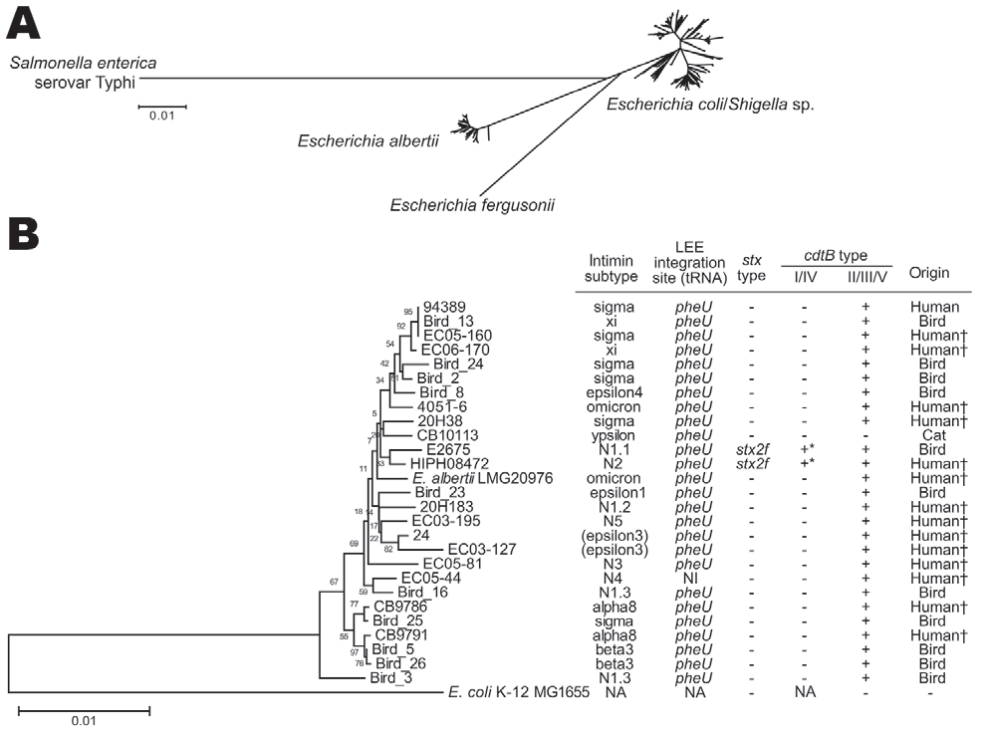
Neighbor-joining tree of 179 *eae*-positive *Escherichia coli* and *Escherichia albertii* strains analyzed by multilocus sequence analysis. The tree was constructed with the concatenated partial nucleotide sequences of 7 housekeeping genes (see Technical Appendix for protocol details). A) The whole image of the 179 strains examined and 10 reference strains (*E. coli*/*Shigella* sp., *E. fergusonii,* and *Salmonella enterica* serovar Typhi) is shown. B) Enlarged view of the *E. albertii* lineage and the genetic information of the identified *E. albertii* strains. *E. coli* strain MG1655 and *E. albertii* type strain LMG20976 are included as references. There was no phylogenetic correlation between human and animal isolates. The *cdtB* genes indicated by * are classified as subtype I. The strains indicated by † were isolated from patients with signs and symptoms of gastrointestinal infection. LEE, locus of enterocyte effacement; NI, not identified; NA, not applicable Scale bars indicate amino acid substitutions (%) per site.

To determine the phylogenetic relationships of the strains, we performed multilocus sequencing analysis of 179 strains that were selected from our collection on the basis of intimin subtype and serotype (see Technical Appendix for selection criteria and analysis protocol). Among the 179 strains, 26 belonged to the *E. albertii* lineage (Figure 2). The 26 *E. albertii* strains were from 14 humans (13 from symptomatic patients), 11 birds, and 1 cat. All of the 5 new intimin subtypes were found in the *E. albertii* strains. Intimin subtypes found in other *E. albertii* strains were also rare subtypes found in *E. coli* (*10*). This finding suggests that more previously unknown intimin subtypes may exist in the *E. albertii* population.

We next analyzed the *pheV*, *selC*, and *pheU* loci of the 26 *E. albertii* strains for the presence of LEE elements as described (Technical Appendix). These 3 genomic loci are the known LEE integration sites in *E. coli*. By this analysis, all *E. albertii* strains except 1 (EC05–44) contained the LEE in the *pheU* locus (the integration site in EC05–44 was not identified). This finding indicates that despite the remarkable diversity of intimin subtypes, the LEE elements are preferentially integrated into the *pheU* tRNA gene in *E. albertii*.

Because all *E. albertii* strains isolated so far contained the *cdtB* gene encoding the cytolethal distending toxin B subunit (*8**,**9*), we examined the presence and subtype of the *cdtB* gene as described (Technical Appendix). This analysis revealed that all *E. albertii* strains except 1 (CB10113) possessed the *cdtB* gene belonging to the II/III/V subtype group (Figure 1, panel B); this finding is consistent with published findings (*9*). In addition, 2 strains (E2675 and HIPH08472) each of which was subtype I , possessed a second *cdtB* gene, (Figure 1, panel B).

We used PCR to further investigate the presence of Shiga toxin genes (*stx*) and their variants (Technical Appendix) and found that 2 *E. albertii* strains possessed the *stx2f* gene (Figure 2, panel B). Stx2 production by these strains was confirmed by using a reverse-passive latex agglutination kit (Technical Appendix). The 2 *stx2f*-positive strains were those containing the subtype I *cdtB* gene in addition to the II/III/V subtype group gene: 1 (HIPH08472) was isolated from a patient with diarrhea and the other (E2675) was from a healthy *Corvus* sp. bird (Figure 2).

Last, we examined the phenotypic and biochemical properties of the 26 *E. albertii* strains and compared the results with those obtained in a previous study (*9*) and with those of *E. albertii* type strain LMG20976 (Table 2). To identify features that could discriminate *E. albertii* from *E. coli*, the results were further compared with those of *E. coli* (*11*). Consistent with findings in previous reports (*5**–**7**,**9*), the lack of motility and the inability to ferment xylose and lactose and to produce β-D-glucuronidase were common biochemical properties of *E. albertii* that could be used to discriminate *E. albertii* from *E. coli*, although 1 *E. albertii* strain was positive for lactose fermentation. The inability of *E. albertii* to ferment sucrose has been described as a common feature (*9*); however, a positive reaction to this test was found for 5 (19.2%) *E. albertii* strains. Moreover, approximately half of *E. coli* strains are positive for sucrose fermentation. Thus, the inability to ferment sucrose is not informative. Rather, the inability to ferment dulcitol (all *E. albertii* strains were negative, 60% of *E. coli* strains are positive) may be a useful biochemical property for differentiation.

**Table 2:** Comparison of biochemical properties of *Escherichia* spp. strains

Agent or test	26 *E. albertii* strains (this study)†	*E. albertii* LMG20976 (type strain)	*E. albertii* strains (*9*)	*E. coli* (*11*)†
Indole	96.2	–	100	98
Motility	0	–	0	95
Urea	0	–	0	1
ONPG	88.5	+	ND	ND
MUG	0	–	ND	(+)‡
Citrate	0	–	0	1
Acetate	92.3	+	ND	90
Malonate	0	–	ND	0
H_2_S on triple sugar iron	0	–	ND	1
Voges-Proskauer	0	–	ND	0
Lysine decarboxylase	100	+	100	90
Ornithine decarboxylase	100	+	100	65
Arginine dihydrolase	0	–	0	17
Glucose, acid	100	+	100	100
Glucose, gas	100	+	100	95
Acid from				
Adonitol	0	–	ND	0
l-arabinose	100	+	100	99
Cellobiose	0	–	ND	2
Dulcitol	0	–	ND	60
Myo-inositol	0	–	ND	1
Lactose	3.9	–	0	95
Maltose	88.5	+	ND	95
Mannitol	100	+	100	100
l-rhamnose	0	–	0	0
Salicin	26.9	–	ND	40
d-sorbitol	57.7	–	V	94
Sucrose	19.2	–	0	50
Trehalose	96.2	+	ND	98
d-xylose	0	–	0	95

*ONPG, ortho-Nitrophenyl-β-galactoside; MUG, methylumbelliferyl-β-D-glucuronide; –, negative; +, positive: ND, not determined. †Average (%) of positive strains. ‡Most *E. coli* strains produce β-D-glucuronidase.

## Conclusion

In the current clinical laboratory setting, a substantial number of *E. albertii* strains are misidentified as EPEC or EHEC. Because 13 of the isolates were from patients with signs and symptoms of gastrointestinal infection, *E. albertii* is probably a major enteric human pathogen. In addition, *E. albertii* should be regarded as a potential Stx2f-producing bacterial species, although the clinical significance of Stx2f-producing strains is unknown.

Notable genetic, phenotypic, and biochemical properties of *E. albertii*, which were identified by analyzing the confirmed *E. albertii* strains, are 1) possession of intimin subtypes rarely or previously undescribed in *E. coli*, 2) possession of the II/III/V subtype group *cdtB* gene, 3) LEE integration into the *pheU* tRNA gene, 4) nonmotility, and 5) inability to ferment xylose, lactose, and dulcitol (but not sucrose) and to produce β-D-glucuronidase. These properties could be useful for facilitating identification of *E. albertii* strains in clinical laboratories, which would in turn improve understanding of the clinical significance and the natural host and niche of this newly recognized pathogen. In this regard, however, current knowledge of the genetic and biological properties of *E. albertii* might be biased toward a certain group of *E. albertii* strains because, even with this study, only a limited number of strains have been analyzed. To more precisely understand the properties of *E. albertii* as a species, further analysis of more strains from various sources is necessary.

## Supplementary Material

Technical Appendix The 275 bacterial strains used were isolated in the laboratories participating in this study or from strain stocks from each laboratory.
