# Supplementary material for: Clinical Significance of Escherichia albertii
Source: Emerg Infect Dis. 2012 Mar;18(3):488–92. doi: 10.3201/eid1803.111401 (PMC3309589; doi:10.3201/eid1803.111401)
Supplement: Technical Appendix — The 275 bacterial strains used were isolated in the laboratories participating in this study or from strain stocks from each laboratory. [file 11-1401-Techapp_18p.pdf]

Article DOI: http://dx.doi.org/10.3201/eid1803.111401

Clinical Significance of *Escherichia albertii*

Technical Appendix

Bacterial strains, growth conditions, and DNA extraction

The 275 bacterial strains used were isolated in the laboratories participating in this study or from strain stocks from each laboratory. The sources of their isolation and other strain information are summarized in Technical Appendix Table 1. In brief, the 275 strains were isolated in Japan, Brazil, Germany, and Belgium. All of the strains had been originally identified as EPEC or EHEC. Among the 193 human isolates, 154 were isolated from patients with the clinical symptoms of a gastrointestinal infection, such as diarrhea (bloody or non-bloody), abdominal pain, vomiting, and fever. As for the remaining 39 human isolates, we confirmed that 7 were from asymptomatic carriers, while clinical records on the others were not available. The 76 animal isolates were mainly from wild birds, which were found dead due to unknown reasons and thus subjected to laboratory examinations, and healthy pigs; these isolates included several strains from other domestic and wild animals. The environmental strains were isolated from sand pit courts at elementary schools, parks, and shrines.) The *E. albertii* type strain LMG20976 was provided by RIKEN BioResource Center (Ibaraki, Japan). Bacterial cells were grown aerobically at 37°C in Luria-Bertani (LB) medium or on LB agar. Bacterial DNA used as template DNA for PCR was prepared by the alkaline-boiling method as described previously (*1*).

Sequence-based intimin subtyping

DNA sequences of the entire *eae* genes were determined as described by Lacher *et al.* (*2*). Briefly, the 5′ half of the gene and its upstream region were amplified by PCR using the cesT-F9/eae-F1 primer pair and KAPATaq (NIPPON Genetics, Tokyo, Japan), and the 3′ half and the downstream region were amplified using the eae-R3/escD-R1 primer pair. Amplicons were sequenced with the primers used for PCR amplification on the ABI 3710 autosequencer (Life Technologies Corporation, CA). To fully sequence the 3′ half, an additional sequence primer

(1669-1688) was used. Primer sequences and amplification conditions are listed in Technical Appendix Table 2.

Predicted amino acid sequences were aligned with those of the reference intimin subtypes listed in Technical Appendix Table 3 by the ClustalW program in MEGA4 (*3*). A phylogenetic tree was constructed with the neighbor-joining algorithm using MEGA4. Poisson correction was used to calculate protein distances. Bootstrap analysis with 1000 replicates was performed to evaluate the significance of internal branches. To define new intimin subtypes, we employed the cutoff value of 95% nucleotide sequence identity (*4*).

Multi-locus sequence (MLS) analysis

To determine the phylogenetic relationships of the *eae*-positive strains, we performed MLS analysis. For this analysis, we selected one or two representative strains for each intimin subtype. When different serotypes were found within an intimin subtype, we selected one or two strains for each serotype; thus, 179 strains were analyzed in total.

MLS analysis was performed using the nucleotide sequences of 7 housekeeping genes (*adk*, *fumC*, *gyrB*, *icd*, *mdh*, *purA*, and *recA*). Target genes were amplified and sequenced according to the protocol provided from the UCC Web site (http://mlst.ucc.ie/). Using the concatenated nucleotide sequences of the 7 genes and the maximum composite likelihood model, a neighbor-joining tree was constructed using MEGA4 software. EcoR collection strains (*5*) and genome-sequenced *E. coli*, *E. fergusonii*, *E. albertii*, *Shigella* sp., and *Salmonella enterica* serovar Typhi strains were included in a phylogenetic representation.

PCR detection and sequencing of the *stx* and *cdtB* genes

PCR screening was performed for the genes for Shiga toxins 1 and 2 (*stx1*, *stx2* and *stx2*-variants) and the B subunit of cytolethal-distending toxin (*cdtB*). All primers and PCR conditions used for this screening are shown in Technical Appendix Table 2. PCR amplification was performed using KAPATaq Extra DNA polymerase (KAPA Biosystems, Inc., MA). Subtypes and phylogenetic relationships of the *cdtB* genes were determined by direct sequencing of the amplicons on the ABI 3710 autosequencer using the primers used for PCR amplification.

Detection of Stx production with or without mitomycin C (MMC) induction

The production of Stx2f by *stx2f*-positive *E. albertii* strains was determined by using a reverse-passive latex agglutination kit (VTEC-RPLA; Denka Seiken Co., Ltd., Tokyo, Japan). Bacterial cell were pre-cultured in 1 mL of Casamino Acids-yeast extract (CAYE) broth (Denka Seiken, Tokyo, Japan) overnight with shaking, and then inoculated to adjust OD_{600} = 0.1 into 2mL of fresh CAYE broth and followed by 16 hrs incubation (MMC-). For mitomycin C (MMC; Kyowa Hakko Kirin Co., Ltd., Tokyo, Japan) induction, 0.5 µl of 2 mg/mL MMC solution was add to the 2 mL culture at an hour incubation (final concentration of 50 µg/mL) and followed by 15 hrs incubation. Of the cell suspension after 16 hrs incubation, 1 mL culture was treated with 1 mL of polymyxin B (Sigma-Aldrich Japan, Tokyo, Japan; final concentration of 5,000 U/mL) for 1 hr at 37°C. The solution were centrifuged for 10 min at 9,000 rpm at 4°C and used for VTEC-RPLA assay according to the manufactures instruction. *E. albertii* strain LMG20976 (type strain; *stx*-negative) and strain CB9786 (*stx*-negative) were used as negative controls. EHEC O128:HNM strain EC1463 (*stx2f*-positive) and EHEC O157:H7 strain Sakai (*stx1*- and *stx2*-positive) were used as positive control (*stx2f* and *stx* genes, respectively). The result of this analysis was shown in Technical Appendix Table 4.

Determination of the LEE integration sites

Three integration sites that have so far been identified for the LEE elements in various *E. coli* strains are the *pheV*, *selC*, and *pheU* tRNA gene loci. It is also known that although the gene organization of LEE core regions is highly conserved between strains, accessory regions of highly variable sizes and genetic structures often exist just downstream of the core region (*6*–*8*). In contrast, no or only small accessory regions have been identified upstream of the core region; thus, the genetic structures of the left (upstream) chromosome/LEE junctions are relatively well conserved. Therefore, by employing long-range PCR targeted to the *escR* gene in the LEE core region and chromosomal regions outside of the left chromosome/LEE junctions, we performed a systematic survey of the *pheV*, *selC*, and *pheU* loci of the *eae*-positive strains for the presence of LEE elements.

Long-range PCR screening was performed by using TaKaRa LA Taq polymerase (Takara Bio Inc. Ohtsu, Japan). Each locus was examined by PCR using an inside primer (escR-R) in

combination with outside primers targeted to the genomic regions adjacent to each tRNA gene locus. The outside primers were designed based on the genome sequences of the K-12 strain MG1655 (*9*) and 5 EHEC and EPEC strains (*6*,*10*,*11*). Primer sequences and amplification conditions are listed in Technical Appendix Table 5.

Phenotype and biochemical characterization of *E. albertii* strains

The phenotypic and biochemical properties of the strains identified as *E. albertii* in this study and the *E. albertii* type strain (LMG20976) were examined by conventional methods (*12*). Carbohydrate-fermenting abilities were determined after 7 days of incubation at 37°C in Andrade peptone water (Oxoid, Cambridge, UK) containing one of the following 15 carbohydrates (Wako Pure Chemicals, Osaka, Japan): adonitol, arabinose, cellobiose, dulcitol, glucose, inositol, lactose, maltose, mannitol, rhamnose, salicin, sorbitol, sucrose, trehalose, and xylose. The β-glucuronidase activity was examined using CLIG medium (Kyokuto Pharmaceutical, Tokyo, Japan).

Nucleotide sequence accession numbers

All nucleotide sequences obtained in this study have been deposited into the DDBJ/EMBL/GenBank database. The accession numbers are AB647359-AB647618 (for the *eae* genes), AB647619-AB647655 (for the *cdtB* genes), and AB647656-648908 (for the 7 housekeeping genes [*adk*, *fumC*, *gyrB*, *icd*, *mdh*, *purA*, and *recA*] used for MLS analysis).

References

1. Ooka T, Terajima J, Kusumoto M, Iguchi A, Kurokawa K, Ogura Y, et al. Development of a multiplex PCR-based rapid typing method for enterohemorrhagic *Escherichia coli* O157 strains. J Clin Microbiol. 2009;47:2888–94. PubMed http://dx.doi.org/10.1128/JCM.00792-09

2. Lacher DW, Steinsland H, Whittam TS. Allelic subtyping of the intimin locus (*eae*) of pathogenic *Escherichia coli* by fluorescent RFLP. FEMS Microbiol Lett. 2006;261:80–7. PubMed http://dx.doi.org/10.1111/j.1574-6968.2006.00328.x

3. Tamura K, Dudley J, Nei M, Kumar S. MEGA4: Molecular Evolutionary Genetics Analysis (MEGA) software version 4.0. Mol Biol Evol. 2007;24:1596–9. PubMed http://dx.doi.org/10.1093/molbev/msm092

4. Zhang WL, Kohler B, Oswald E, Beutin L, Karch H, Morabito S, et al. Genetic diversity of intimin genes of attaching and effacing *Escherichia coli* strains. J Clin Microbiol. 2002;40:4486–92. PubMed http://dx.doi.org/10.1128/JCM.40.12.4486-4492.2002

5. Ochman H, Selander RK. Standard reference strains of *Escherichia coli* from natural populations. J Bacteriol. 1984;157:690–3. PubMed

6. Ogura Y, Ooka T, Iguchi A, Toh H, Asadulghani M, Oshima K, et al. Comparative genomics reveal the mechanism of the parallel evolution of O157 and non-O157 enterohemorrhagic *Escherichia coli*. Proc Natl Acad Sci U S A. 2009;106:17939–44. PubMed http://dx.doi.org/10.1073/pnas.0903585106

7. Jores J, Rumer L, Wieler LH. Impact of the locus of enterocyte effacement pathogenicity island on the evolution of pathogenic *Escherichia coli*. Int J Med Microbiol. 2004;294:103–13. PubMed http://dx.doi.org/10.1016/j.ijmm.2004.06.024

8. Müller D, Benz I, Liebchen A, Gallitz I, Karch H, Schmidt MA. Comparative analysis of the locus of enterocyte effacement and its flanking regions. Infect Immun. 2009;77:3501–13. PubMed http://dx.doi.org/10.1128/IAI.00090-09

9. Blattner FR, Plunkett G, Bloch CA, Perna NT, Burland V, Riley M, et al. The complete genome sequence of *Escherichia coli* K-12. Science. 1997;277:1453–62. PubMed http://dx.doi.org/10.1126/science.277.5331.1453

10. Hayashi T, Makino K, Ohnishi M, Kurokawa K, Ishii K, Yokoyama K, et al. Complete genome sequence of enterohemorrhagic *Escherichia coli* O157:H7 and genomic comparison with a laboratory strain K-12. DNA Res. 2001;8:11–22. PubMed http://dx.doi.org/10.1093/dnares/8.1.11

11. Iguchi A, Thomson NR, Ogura Y, Saunders D, Ooka T, Henderson IR, et al. Complete genome sequence and comparative genome analysis of enteropathogenic *Escherichia coli* O127:H6 strain E2348/69. J Bacteriol. 2009;191:347–54. PubMed http://dx.doi.org/10.1128/JB.01238-08

12. Ewing WH. The Genus Escherichia. In: Ewing WH, Edwards PR editors. Identification of *Enterobacteriaceae*, 4th ed. New York: Elsevier Science Publishing; 1986. p. 93–134.

Technical Appendix Table 1. Detailed information of the strains used in this study

Serotype†	strain name	year of isolate	origin	countries	symptoms or notes	intimin subtypes	LEE integration sites (tRNA gene)	presence or absence		MLST analysis	References
								stx1&2&2f	cdtB		
O51:H40	EC06-71	2006	human	Japan	symptomatic	theta	NT	-	-	Y	this study
O40/33:H34	EC06-80	2006	human	Japan	symptomatic (diarrhea, abdominal pain, fever)	(lambda)	selC	-	-	Y	this study
O88:H8	EC06-90	2006	human	Japan	symptomatic	iota1	selC	-	+	Y	this study
O51:H40	EC06-118	2006	human	Japan	symptomatic (diarrhea, abdominal pain)	theta	NT	-	-	Y	this study
OUT:H34	EC06-119	2006	human	Japan	symptomatic (diarrhea, abdominal pain)	iota1	selC	-	-	Y	this study
O175:NM	EC06-170	2006	human	Japan	symptomatic	xi	pheU	-	+	Y‡	this study
O55:H6	18H89	2006	human	Japan	symptomatic	iota1	selC	-	+	Y	this study
O145:H34	19H198	2007	human	Japan	symptomatic (diarrhea, abdominal pain, fever)	iota1	selC	-	-	Y	this study
O23:H8	19H226	2007	human	Japan	symptomatic (diarrhea, abdominal pain)	theta	NT	-	-	Y	this study
O65:NM	20H38	2008	human	Japan	symptomatic (diarrhea)	sigma	pheU	-	+	Y‡	this study
O152/115:NM	20H183	2008	human	Japan	symptomatic (diarrhea)	N1.3	pheU	-	+	Y‡	this study
O113:H19	20H186	2008	human	Japan	symptomatic (diarrhea)	epsilon2	selC	-	+	Y	this study
O114:H19	20H215	2008	human	Japan	symptomatic (diarrhea)	epsilon2	selC	-	+	Y	this study
O101:NM	20H250	2009	human	Japan	symptomatic (diarrhea, fever)	(iota2)	pheU	-	-	Y	this study
O101:NM	21H147	2009	human	Japan	symptomatic (diarrhea)	iota2	pheU	-	+	Y	this study
O21:H8	EC01-376	2001	environment	Japan	sand pit court	theta	selC	-	+	Y	this study
O66:H21	EC01-380	2001	environment	Japan	sand pit court	theta	NT	-	+	Y	this study
O142:H34	EC01-383	2001	environment	Japan	sand pit court	alpha1	selC	-	-	Y	this study
OUT:H21	EC01-386	2001	environment	Japan	sand pit court	theta	NT	-	+	Y	this study
O51:H49	EC01-403	2001	environment	Japan	sand pit court	alpha1	selC	-	+	Y	this study
OUT:H34	EC01-406	2001	environment	Japan	sand pit court	alpha2	selC	-	-	Y	this study
O128:NM	EC01-460	2001	human	Japan	symptomatic (diarrhea, abdominal pain, fever)	beta1	NT	-	-	Y	this study
O5:NM	EC03-71	2003	human	Japan	symptomatic (diarrhea, bloody stool, fever, abdominal pain)	beta1	NT	stx1&stx2	-	Y	this study
OUT:H34	EC03-82	2003	human	Japan	symptomatic (diarrhea, bloody stool, fever, abdominal pain)	iota1	selC	-	-	Y	this study
O51:H40	EC03-93	2003	human	Japan	symptomatic (diarrhea, abdominal pain)	epsilon1	NT	-	-	Y	this study
OUT:H6	EC03-126	2003	human	Japan	symptomatic (diarrhea)	beta2	selC	-	-	Y	this study

Serotype†	strain name	year of isolate	origin	countries	symptoms or notes	intimin subtypes	LEE integration sites (tRNA gene)	presence or absence		MLST analysis	References
								stx1&2&2f	cdtB		
O181:NM	EC03-127	2003	human	Japan	symptomatic (diarrhea)	(epsilon3)	pheU	-	+	Y‡	this study
O180:NM	EC03-144	2003	human	Japan	asymptomatic carrier	rho	pheU	-	-	Y	this study
O153:H21	EC03-152	2003	human	Japan	symptomatic (diarrhea, fever)	theta	NT	-	-	Y	this study
OUT:NM	EC03-195	2003	human	Japan	symptomatic	N5	pheU	-	+	Y‡	this study
OUT:H21	EC03-207	2003	animal	Japan	asymptomatic	theta	selC	-	-	Y	this study
OUT:H21	EC03-211	2003	animal	Japan	asymptomatic	theta	NT	-	-	Y	this study
OUT:H6	EC03-224	2003	animal	Japan	asymptomatic	beta2	selC	-	-	Y	this study
OUT:H34	EC04-81	2004	human	Japan	symptomatic (abdominal pain, vomitting)	iota1	selC	-	-	Y	this study
O88:H25	EC04-258	2004	human	Japan	symptomatic (diarrhea, vomitting)	epsilon2	selC	-	-	Y	this study
O21:H6	EC04-268	2004	human	Japan	symptomatic (diarrhea)	alpha2	selC	-	-	Y	this study
O117:H21	EC04-311	2004	human	Japan	symptomatic (diarrhea, vomitting)	theta	NT	-	-	Y	this study
O152:H38	EC04-437	2004	human	Japan	symptomatic (abdominal pain)	epsilon1	NT	-	-	Y	this study
OUT:H2	EC04-500	2004	human	Japan	symptomatic (diarrhea)	beta1	NT	-	-	Y	this study
OUT:H34	EC04-569	2004	human	Japan	symptomatic (diarrhea, vomitting)	iota1	selC	-	-	Y	this study
OUT:H21	EC04-572	2004	human	Japan	symptomatic (diarrhea, abdominal pain)	beta1	NT	-	-	Y	this study
O49:H10	EC04-588	2004	human	Japan	symptomatic	(kappa)	selC	-	-	Y	this study
OUT:NM	EC05-44	2005	human	Japan	symptomatic	N4	NT	-	+	Y‡	this study
O129/13:H11	EC05-63	2005	human	Japan	symptomatic (diarrhea)	omicron	pheU	-	-	Y	this study
O108:H40	EC05-66	2005	human	Japan	symptomatic (diarrhea, abdominal pain)	epsilon1	NT	-	-	Y	this study
OUT:NM	EC05-81	2005	human	Japan	symptomatic	N3	pheU	-	+	Y‡	this study
O70:H11	EC05-86	2005	human	Japan	asymptomatic carrier	epsilon1	NT	-	-	Y	this study
O128:NM	EC05-93	2005	human	Japan	symptomatic	beta1	NT	-	-	N	this study
OUT:H34	EC05-94	2005	human	Japan	symptomatic	alpha2	selC	-	-	N	this study
O71:H49	EC05-95	2005	human	Japan	asymptomatic carrier	kappa	selC	-	-	Y	this study
O10:NM	EC05-134	2005	human	Japan	symptomatic (diarrhea, abdominal pain)	iota1	selC	-	-	Y	this study
OUT:NM	EC05-160	2005	human	Japan	symptomatic	sigma	pheU	-	+	Y‡	this study
OUT:49	EC05-165	2005	human	Japan	symptomatic	alpha1	selC	-	-	Y	this study
OUT:H4	EC05-171	2005	human	Japan	symptomatic	omicron	pheU	-	-	Y	this study
O171:H19	12H133	2000	human	Japan	NI	epsilon2	selC	-	-	Y	this study
O119:H2	12H377	2000	human	Japan	NI	beta1	NT	-	-	Y	this study
O2:H49	17H285	2005	human	Japan	symptomatic	iota1	selC	-	-	Y	this study
OUT:HND	93010	1993.6.18	human	Japan	symptomatic (diarrhea)	mu	selC	-	-	Y	this study
OUT:H40	94037	1994.6.29	human	Japan	symptomatic (diarrhea, fever)	(eta2)	selC	-	+	Y	this study
OUT:HND	94046-2	1994.7.25	human	Japan	symptomatic (bloody diarrhea)	epsilon2	selC	-	-	Y	this study

Serotype†	strain name	year of isolate	origin	countries	symptoms or notes	intimin subtypes	LEE integration sites (tRNA gene)	presence or absence		MLST analysis	References
								stx1&2&2f	cdtB		
OUT:H7	94064	1994.9.19	human	Japan	symptomatic (diarrhea)	theta	NT	-	-	Y	this study
O86a:HND	94308	1994.6.25	human	Japan	NI	iota1	selC	-	-	Y	this study
O55:H7	94327	1994.7.12	human	Japan	NI	gamma1	selC	-	-	Y	this study
O26:H21	94358	1994.8.6	human	Japan	NI	theta	NT	-	-	N	this study
OUT:HND	94368	1994.8.16	human	Japan	NI	theta	NT	-	-	Y	this study
OUT:HND	94389	1994.9.8	human	Japan	NI	sigma	pheU	-	+	Y‡	this study
OUT:HNM	94414	1994.10.12	human	Japan	NI	theta	NT	-	-	Y	this study
O55:H7	95012	1995.5.11	human	Japan	symptomatic (diarrhea)	gamma1	selC	-	-	N	this study
O119:H2	95028	1995.6.12	human	Japan	symptomatic (diarrhea)	beta1	NT	-	-	Y	this study
OUT:HND	95032	1995.6.16	human	Japan	symptomatic (diarrhea)	iota1	selC	-	-	Y	this study
O26:HNM	95036-2	1995.6.18	human	Japan	symptomatic (diarrhea)	beta1	NT	-	-	Y	this study
OUT:HND	95037	1995.6.19	human	Japan	symptomatic (diarrhea, fever)	epsilon2	selC	-	-	Y	this study
OUT:HND	95301	1995.5.9	human	Japan	symptomatic (bloody diarrhea, fever, abdominal pain)	iota1	selC	-	-	Y	this study
O15:HND	95473	1995.10.27	human	Japan	NI	beta1	NT	-	-	Y	this study
O153:H7	960064	1996.7.2	human	Japan	symptomatic (bloody diarrhea)	beta1	selC	-	-	Y	this study
O26:HNM	960067	1996.7.5	human	Japan	symptomatic (diarrhea)	beta1	NT	-	-	N	this study
O126:HND	960134	1996.8.2	human	Japan	symptomatic (diarrhea, fever)	iota1	selC	-	-	Y	this study
OUT:HND	960135	1996.8.2	human	Japan	symptomatic (diarrhea, fever)	gamma1	selC	-	-	Y	this study
O20:HND	960175	1996.8.23	human	Japan	symptomatic (diarrhea, fever)	beta2	selC	-	-	N	this study
OUT:HND	960185	1996.8.29	human	Japan	symptomatic (bloody diarrhea)	beta2	selC	-	-	Y	this study
OUT:HND	960192	1996.8.31	human	Japan	symptomatic (diarrhea, fever)	gamma1	selC	-	-	Y	this study
O115:HND	960719	1996.9.30	human	Japan	symptomatic (diarrhea)	theta	NT	-	-	N	this study
O20:H6	960241	1996.3.18	human	Japan	NI	beta2	selC	-	-	Y	this study
O115:HND	960242	1996.3.19	human	Japan	symptomatic (bloody stool, abdominal pain, vomitting)	beta2	selC	-	-	Y	this study
O15:H2	960261	1996.5.13	human	Japan	symptomatic (bloody stool, fever, abdominal pain)	beta1	NT	-	-	Y	this study
OUT:HND	960296	1996.6.25	human	Japan	NI	iota1	selC	-	-	Y	this study
OUT:HND	960337	1996.7.15	human	Japan	NI	zeta3	selC	-	-	Y	this study
NI	960349	1996.7.19	human	Japan	NI	zeta3	selC	-	-	Y	this study
OUT:HND	960446	1996.8.8	human	Japan	NI	beta2	selC	-	-	Y	this study
O26:HND	960462	1996.8.29	human	Japan	NI	kappa	selC	-	-	Y	this study
OUT:HND	960468	1996.9.6	human	Japan	NI	eta2	selC	-	-	Y	this study
O26:HNM	960496	1996.10.14	human	Japan	NI	beta1	selC	-	-	N	this study
O119:HNM	97054-1	1997.6.13	human	Japan	symptomatic (diarrhea,	theta	NT	-	-	Y	this study

Serotype†	strain name	year of isolate	origin	countries	symptoms or notes	intimin subtypes	LEE integration sites (tRNA gene)	presence or absence		MLST analysis	References
								stx1&2&2f	cdtB		
					abdominal pain, fever)						
OUT:HND	97105	1997.8.8	human	Japan	symptomatic (diarrhea, vomitting)	beta1	pheU	-	-	Y	this study
NI	97144	1997.9.11	human	Japan	symptomatic (bloody diarrhea)	theta	NT	-	-	Y	this study
O26:HNM	97207	1997.11.14	human	Japan	symptomatic (bloody diarrhea)	beta1	NT	-	-	N	this study
O55:H7	97214	1997.11	human	Japan	symptomatic	gamma1	selC	-	-	N	this study
O55:HND	97253-2	1997.12.20	human	Japan	symptomatic (bloody diarrhea)	gamma1	selC	-	-	N	this study
O157:HND	97255	1997.12.17	human	Japan	symptomatic (diarrhea)	alpha1	selC	-	-	Y	this study
O146:H21	97603	1997.6.9	human	Japan	NI	theta	NT	-	-	Y	this study
O167:HND	97604	1997.6.10	human	Japan	NI	beta1	NT	-	-	Y	this study
O168:HND	97650	1997.6.21	human	Japan	NI	gamma1	selC	-	-	Y	this study
O128:HND	97651	1997.6.21	human	Japan	symptomatic (diarrhea, abdominal pain, vomitting, fever)	beta1	NT	-	-	N	this study
OUT:HND	97674-2	1997.6.30	human	Japan	symptomatic (bloody stool, abdominal pain)	epsilon2	selC	-	-	Y	this study
O128:H2	97756	1997.7,19	human	Japan	symptomatic (diarrhea)	beta1	NT	-	-	Y	this study
OUT:HND	97845	1997.8.8	human	Japan	NI	iota1	selC	-	-	Y	this study
O126:H6	97846	1997.8.8	human	Japan	symptomatic (diarrhea, fever)	alpha2	selC	-	-	Y	this study
O146:H7	97938	1997.9.1	human	Japan	symptomatic (bloody diarrhea, abdominal pain)	epsilon1	NT	-	-	Y	this study
OUT:HND	971107	1997.12.20	human	Japan	symptomatic (diarrhea)	epsilon1	NT	-	-	Y	this study
O55:H7	98078	1998.5.26	human	Japan	NI	beta1	pheU	-	-	Y	this study
O55:H7	98117	1998.6.24	human	Japan	symptomatic (diarrhea)	gamma1	selC	-	-	N	this study
OUT:HND	98257	1998.10.28	human	Japan	symptomatic (bloody diarrhea)	theta	NT	-	-	Y	this study
O153:H7	98275	1998.11.6	human	Japan	NI	beta1	NT	-	-	Y	this study
O20:HNM	98288	1998.11.30	human	Japan	symptomatic (diarrhea, vomitting, fever)	beta2	selC	-	-	Y	this study
O55:H7	99600	1999.7.8	human	Japan	symptomatic (diarrhea, abdominal pain)	gamma1	selC	-	-	N	this study
OUT:H2	99622	1999.8.11	human	Japan	symptomatic (diarrhea, abdominal pain)	beta1	selC	-	-	Y	this study
O119:HNM	99638	1999.8.23	human	Japan	symptomatic (diarrhea)	beta1	selC	-	-	Y	this study
OUT:HND	99066	1999.8.3	human	Japan	symptomatic (diarrhea)	iota1	selC	-	-	Y	this study
O127a:H40	99067	1999.8.2	human	Japan	symptomatic (diarrhea, abdominal pain)	theta	NT	-	-	Y	this study
O153:H7	99674	1999.9.30	human	Japan	symptomatic (diarrhea)	beta1	selC	-	-	N	this study
O119:HNM	99697	1999.10.17	human	Japan	symptomatic (bloody diarrhea, fever)	beta1	pheU	-	-	N	this study
O26:HNM	540	2000.4	human	Japan	symptomatic (diarrhea)	beta1	NT	-	-	N	this study

Serotype†	strain name	year of isolate	origin	countries	symptoms or notes	intimin subtypes	LEE integration sites (tRNA gene)	presence or absence		MLST analysis	References
								stx1&2&2f	cdtB		
O55:H7	544	2000.4.21	human	Japan	symptomatic (bloody stool)	gamma1	selC	-	-	N	this study
OUT:HND	24	2000.4.14	human	Japan	symptomatic (diarrhea, abdominal pain)	(epsilon3)	pheU	-	+	Y‡	this study
O55:H7	594	2000.7	human	Japan	symptomatic (diarrhea, abdominal pain, vomitting)	gamma1	selC	-	-	N	this study
O128:H2	595	2000.7.22	human	Japan	symptomatic (diarrhea, abdominal pain, fever)	beta1	NT	-	-	N	this study
O26:H11	608	2000.8.4	human	Japan	symptomatic (diarrhea, fever)	beta1	NT	-	-	N	this study
O128:H2	618	2000.8.10	human	Japan	symptomatic (diarrhea, abdominal pain)	beta1	pheV	-	-	N	this study
O26:HUT	626	2000.8	human	Japan	symptomatic (diarrhea, vomitting)	theta	NT	-	-	Y	this study
O119:HNM	629	2000.8.25	human	Japan	NI	beta1	pheU	-	-	N	this study
O159:HNM	664	2000.10.16	human	Japan	symptomatic (diarrhea)	theta	NT	-	-	Y	this study
O128:H2	674	2000.10.27	human	Japan	NI	beta1	NT	-	-	N	this study
OUT:HND	80	2000.10.6	human	Japan	symptomatic (bloody stool)	theta	NT	-	-	Y	this study
O126:H19	1558	2001.6.16	human	Japan	symptomatic (diarrhea, abdominal pain, vomitting)	iota1	selC	-	-	Y	this study
O124:H16	01601-2	2001.7.28	human	Japan	NI	rho	pheU	-	-	Y	this study
OUT:HNM	1065	2001.7	human	Japan	symptomatic (diarrhea)	theta	NT	-	-	Y	this study
O128:H2	1614	2001.8.16	human	Japan	NI	beta1	NT	-	-	N	this study
O20:H6	1086	2001.8.17	human	Japan	symptomatic (diarrhea, vomitting)	beta2	selC	-	-	N	this study
O114:H19	1631	2001.9.4	human	Japan	NI	epsilon2	selC	-	-	Y	this study
OUT:H21	1121	2001.10.10	human	Japan	symptomatic (diarrhea)	theta	selC	-	-	Y	this study
OUT:H6	1128	2001.10.18	human	Japan	symptomatic (bloody stool, abdominal pain, fever)	beta2	selC	-	-	Y	this study
O55:H7	1687	2001.11	human	Japan	symptomatic (bloody stool, fever)	gamma1	selC	-	-	N	this study
O55:H7	01689-1	2001.11.27	human	Japan	symptomatic (diarrhea, fever)	gamma1	selC	-	-	N	this study
O119:HNM	1691	2001.11.29	human	Japan	symptomatic (diarrhea, abdominal pain)	beta1	pheU	-	-	N	this study
O119:HNM	2528	2002.4.8	human	Japan	symptomatic (bloody stool)	beta1	pheU	-	-	N	this study
OUT:HND	2059	2002.6.5	human	Japan	symptomatic (diarrhea, vomitting, fever)	alpha2	selC	-	-	Y	this study
O55:H7	2075	2002.6.27	human	Japan	symptomatic (diarrhea)	beta1	pheV	-	-	Y	this study
O128:H2	2584	2002.7.13	human	Japan	symptomatic (diarrhea, fever)	beta1	pheV	-	-	N	this study

Serotype†	strain name	year of isolate	origin	countries	symptoms or notes	intimin subtypes	LEE integration sites (tRNA gene)	presence or absence		MLST analysis	References
								stx1&2&2f	cdtB		
O128:HND	2595	2002.7.22	human	Japan	symptomatic (diarrhea, fever)	beta1	pheV	-	-	N	this study
O55:H7	2604	2002.8.3	human	Japan	NI	gamma1	selC	-	-	N	this study
O55:H7	2612	2002.8.12	human	Japan	NI	gamma1	selC	-	-	N	this study
O55:H7	2626	2002.8.26	human	Japan	symptomatic (diarrhea, fever)	gamma1	selC	-	-	N	this study
OUT:HND	2184	2002.11.25	human	Japan	symptomatic (diarrhea, vomitting)	beta2	selC	-	-	Y	this study
O55:H7	3114	2003.7.30	human	Japan	symptomatic (diarrhea, fever)	gamma1	selC	-	-	N	this study
O26:H-	3641	2003.7.	human	Japan	symptomatic (diarrhea)	beta1	NT	-	-	N	this study
OUT:HND	3124	2003.8.5	human	Japan	symptomatic (bloody stool, abdominal pain, fever)	epsilon2	selC	-	-	Y	this study
O55:H7	3649	2003.8.1	human	Japan	symptomatic (diarrhea)	gamma1	selC	-	-	N	this study
O128:H2	3705	2003.8.29	human	Japan	symptomatic (diarrhea)	beta1	NT	-	-	N	this study
O26:HNM	03706-2	2003.9.3	human	Japan	NI	beta1	NT	-	-	N	this study
O55:H7	4676	2004.9.17	human	Japan	symptomatic (diarrhea)	iota1	selC	-	-	N	this study
O153:HND	4679	2004.9.24	human	Japan	symptomatic (diarrhea)	beta1	NT	-	-	N	this study
O142:HUT	6592	2006.6.2	human	Japan	symptomatic (diarrhea, abdominal pain)	alpha1	selC	-	-	Y	this study
O55:H7	7575	2007.5.16	human	Japan	symptomatic (diarrhea)	gamma1	selC	-	-	N	this study
O55:H7	7675	2007.7.23	human	Japan	symptomatic (diarrhea)	gamma1	selC	-	-	N	this study
O55:H7	7693	2007.8.1	human	Japan	symptomatic (diarrhea)	gamma1	selC	-	-	N	this study
O55:H7	7707	2007.8.8	human	Japan	symptomatic (diarrhea)	gamma1	selC	-	-	N	this study
O119:H-	7753	2007.8.30	human	Japan	symptomatic (diarrhea)	beta1	pheU	-	-	N	this study
O124:HUT	7852	2007.10.23	human	Japan	symptomatic (diarrhea)	theta	NT	-	-	Y	this study
O26:H-	7857	2007.10.29	human	Japan	NI	beta1	NT	-	-	N	this study
O74:HND	7871	2007.11.9	human	Japan	symptomatic (diarrhea)	iota1	selC	-	-	Y	this study
O103:H-	7929	2007.12.4	human	Japan	symptomatic (bloody stool)	beta1	NT	-	-	Y	this study
O103	NIAH_Por_1	2007	pig	Japan	rectal swab, healthy	beta1	NT	-	-	Y	this study
OUT	NIAH_Por_2	2007	pig	Japan	rectal swab, healthy	gamma1	selC	-	-	N	this study
OUT	NIAH_Por_4	2007	pig	Japan	rectal swab, healthy	gamma1	selC	-	-	N	this study
O49	NIAH_Por_5	2007	pig	Japan	rectal swab, healthy	kappa	selC	-	-	Y	this study
O117	NIAH_Por_8	2007	pig	Japan	rectal swab, healthy	theta	pheV	-	-	N	this study
OUT	NIAH_Por_9	2007	pig	Japan	rectal swab, healthy	gamma1	selC	-	-	N	this study
O88	NIAH_Por_10	2007	pig	Japan	rectal swab, healthy	beta1	pheU	-	-	Y	this study
O76	NIAH_Por_11	2007	pig	Japan	rectal swab, healthy	theta	NT	-	-	N	this study
O145	NIAH_Por_12	2007	pig	Japan	rectal swab, healthy	gamma1	selC	-	-	Y	this study
OUT	NIAH_Por_13	2007	pig	Japan	rectal swab, healthy	xi	NT	-	-	N	this study
O26	NIAH_Por_14	2007	pig	Japan	rectal swab, healthy	xi	NT	-	-	Y	this study
O2	NIAH_Por_15	2007	pig	Japan	rectal swab, healthy	iota1	selC	-	-	Y	this study
O145	NIAH_Por_16	2007	pig	Japan	rectal swab, healthy	gamma1	selC	-	-	N	this study
O53	NIAH_Por_17	2007	pig	Japan	rectal swab, healthy	gamma1	selC	-	-	Y	this study

Serotype†	strain name	year of isolate	origin	countries	symptoms or notes	intimin subtypes	LEE integration sites (tRNA gene)	presence or absence		MLST analysis	References
								stx1&2&2f	cdtB		
O117	NIAH_Por_18	2007	pig	Japan	rectal swab, healthy	theta	pheV	-	-	N	this study
O172	NIAH_Por_19	2007	pig	Japan	rectal swab, healthy	gamma1	selC	-	-	Y	this study
O172	NIAH_Por_20	2007	pig	Japan	rectal swab, healthy	gamma1	selC	-	-	N	this study
O117	NIAH_Por_21	2007	pig	Japan	rectal swab, healthy	theta	pheV	-	-	N	this study
O117	NIAH_Por_22	2007	pig	Japan	rectal swab, healthy	theta	pheV	-	-	N	this study
O117	NIAH_Por_23	2007	pig	Japan	rectal swab, healthy	theta	pheV	-	-	N	this study
O172	NIAH_Por_24	2007	pig	Japan	rectal swab, healthy	gamma1	selC	-	-	N	this study
O156	NIAH_Por_25	2007	pig	Japan	rectal swab, healthy	theta	NT	-	-	N	this study
O98	NIAH_Por_26	2007	pig	Japan	rectal swab, healthy	theta	pheU	-	-	N	this study
OUT	NIAH_Por_27	2007	pig	Japan	rectal swab, healthy	xi	pheU	-	-	N	this study
O49	NIAH_Por_33	2007	pig	Japan	rectal swab, healthy	kappa	selC	-	-	N	this study
OUT	NIAH_Por_34	2007	pig	Japan	rectal swab, healthy	theta	pheV	-	-	N	this study
O172	NIAH_Por_35	2007	pig	Japan	rectal swab, healthy	gamma1	selC	-	-	N	this study
O8	NIAH_Por_36	2007	pig	Japan	rectal swab, healthy	gamma1	selC	-	-	Y	this study
O145	NIAH_Por_37	2007	pig	Japan	rectal swab, healthy	gamma1	selC	-	-	N	this study
O145	NIAH_Por_38	2007	pig	Japan	rectal swab, healthy	gamma1	selC	-	-	N	this study
O53	NIAH_Por_40	2007	pig	Japan	rectal swab, healthy	gamma1	selC	-	-	N	this study
O71	NIAH_Bird_1	2003	Bird	Japan	feces, *Treron sieboldii*	kappa	selC	-	-	Y	this study
OUT	NIAH_Bird_2	2002	Bird	Japan	feces, *Sturnus cineraceus*	sigma	pheU	-	+	Y‡	this study
O115	NIAH_Bird_3	2004	Bird	Japan	feces, *Puffinus tenuirostris*	N1.1	pheU	-	+	Y‡	this study
O137	NIAH_Bird_4	2004	Bird	Japan	feces, *Passer montanus*	beta2	selC	-	-	Y	this study
O128	NIAH_Bird_5	2004	Bird	Japan	feces, *Puffinus tenuirostris*	beta3	pheU	-	+	Y‡	this study
O117	NIAH_Bird_6	2004	Bird	Japan	feces, *Hirundo rustica*	mu	selC	-	-	Y	this study
O117	NIAH_Bird_7	2004	Bird	Japan	feces, *Passer montanus*	mu	selC	-	-	N	this study
O64	NIAH_Bird_8	2004	Bird	Japan	feces, *Egretta garzetta*	epsilon4	pheU	-	+	Y‡	this study
O21	NIAH_Bird_9	2004	Bird	Japan	feces, *Hirundo rustica*	beta1	NT	-	-	Y	this study
O81	NIAH_Bird_10	2004	Bird	Japan	feces, *Anas poecilorhyncha*	beta2	selC	-	+	Y	this study
O55	NIAH_Bird_11	2005	Bird	Japan	feces, *Emberiza cioides*	theta	NT	-	-	N	this study
O2	NIAH_Bird_12	2005	Bird	Japan	feces, *Sturnus cineraceus*	beta1	NT	-	-	Y	this study
OUT	NIAH_Bird_13	2005	Bird	Japan	feces, *Hypsipetes amaurotis*	xi	pheU	-	+	Y‡	this study
O55	NIAH_Bird_15	2005	Bird	Japan	feces, *Cyanopica cyana*	theta	NT	-	-	N	this study
O103	NIAH_Bird_16	2005	Bird	Japan	feces, *Passer montanus*	N1.1	pheU	-	+	Y‡	this study
O55	NIAH_Bird_17	2005	Bird	Japan	feces, *Streptopelia orientalis*	theta	NT	-	-	N	this study
O120	NIAH_Bird_18	2005	Bird	Japan	feces, *Anas strepera*	pi	selC	-	-	N	this study
O132	NIAH_Bird_19	2005	Bird	Japan	feces, *Columba livia*	alpha2	selC	-	-	N	this study
O132	NIAH_Bird_20	2005	Bird	Japan	feces, *Columba livia*	alpha2	selC	-	-	Y	this study
O50	NIAH_Bird_21	2005	Bird	Japan	feces, *Streptopelia orientalis*	alpha1	selC	-	-	Y	this study

Serotype†	strain name	year of isolate	origin	countries	symptoms or notes	intimin subtypes	LEE integration sites (tRNA gene)	presence or absence		MLST analysis	References
								stx1&2&2f	cdtB		
O171	NIAH_Bird_22	2005	Bird	Japan	feces, *Phalacrocorax carbo*	epsilon2	selC	-	-	Y	this study
O58	NIAH_Bird_23	2006	Bird	Japan	feces, *Phalacrocorax carbo*	epsilon1	pheU	-	+	Y‡	this study
O147	NIAH_Bird_24	2006	Bird	Japan	feces, *Cyanopica cyana*	sigma	pheU	-	+	Y‡	this study
O8	NIAH_Bird_25	2006	Bird	Japan	feces, *Passer montanus*	sigma	pheU	-	+	Y‡	this study
O128	NIAH_Bird_26	2006	Bird	Japan	feces, *Hypsipetes amaurotis*	beta3	pheU	-	+	Y‡	this study
O8	NIAH_Bird_27	2006	Bird	Japan	feces, *Phalacrocorax carbo*	beta1	NT	-	-	N	this study
O137	NIAH_Bird_28	2006	Bird	Japan	feces, *Zosterops japonica*	beta2	selC	-	-	N	this study
O56	NIAH_Bird_29	2005	Bird	Japan	foot, *Hypsipetes amaurotis*	beta2	selC	-	-	Y	this study
O55	NIAH_Bird_30	2005	Bird	Japan	foot, *Phalacrocorax carbo*	theta	NT	-	-	N	this study
O132	NIAH_Bird_31	2005	Bird	Japan	foot, *Columba livia*	alpha2	selC	-	-	N	this study
O120	NIAH_Bird_32	2005	Bird	Japan	foot, *Coturnix japonica*	pi	selC	-	-	Y	this study
O110	NIAH_Bird_33	2006	Bird	Japan	foot, *Puffinus tenuirostris*	beta2	selC	-	-	Y	this study
O2	NIAH_Bird_34	2006	Bird	Japan	foot, *Columba livia*	kappa	selC	-	-	Y	this study
O55	NIAH_Bird_35	2006	Bird	Japan	foot, *Hypsipetes amaurotis*	theta	NT	-	-	N	this study
O8	NIAH_Bird_36	2006	Bird	Japan	foot, *Phalacrocorax carbo*	beta1	NT	-	-	Y	this study
O55	NIAH_Bird_37	2006	Bird	Japan	foot, *Sturnus cineraceus*	theta	NT	-	-	N	this study
O55	NIAH_Bird_38	2006	Bird	Japan	foot, *Columba livia*	theta	NT	-	-	N	this study
O103:H2	00E001	2000	human	Japan	symptomatic (diarrhea, abdominal pain, fever)	epsilon1	NT	stx1	-	Y	this study
O150:H11	00E019	2000	human	Japan	symptomatic (diarrhea, bloody stool, abdominal pain, fever)	beta1	pheU	stx1	-	Y	this study
O103:H11	01E015	2001	human	Japan	symptomatic (diarrhea, abdominal pain, fever)	beta1	pheU	stx1	-	Y	this study
O103:H2	02E028	2002	human	Japan	symptomatic (diarrhea, fever)	epsilon1	NT	stx1	-	N	this study
O165:H-	04E077	2000	human	Japan	symptomatic (diarrhea, abdominal pain, fever)	epsilon1	NT	stx2	-	N	this study
O121:H14	06E050	2006	human	Japan	symptomatic (diarrhea, bloody stool)	epsilon1	NT	stx2	-	Y	this study
O103:H2	07E030	2007	human	Japan	symptomatic (diarrhea, bloody stool, abdominal pain)	epsilon1	NT	stx1	-	N	this study
O63:H6	07E033	2000	human	Japan	symptomatic (diarrhea, abdominal pain)	(alpha2)	selC	stx2f	+	Y	this study
O165:H-	07E051	2007	human	Japan	symptomatic (diarrhea, bloody stool, abdominal	epsilon1	NT	stx1&2	-	Y	this study

Serotype†	strain name	year of isolate	origin	countries	symptoms or notes	intimin subtypes	LEE integration sites (tRNA gene)	presence or absence		MLST analysis	References
								stx1&2&2f	cdtB		
					pain)						
O118:H-	07E054	2007	human	Japan	symptomatic (diarrhea, abdominal pain)	beta1	NT	stx1	-	Y	this study
O103:H2	08E011	2008	human	Japan	asymptomatic carrier	epsilon1	NT	stx1	-	N	this study
O103:HUT	08E021	2008	human	Japan	asymptomatic carrier	theta	NT	stx1	-	N	this study
O121:H19	08E027	2008	human	Japan	symptomatic (diarrhea, bloody stool, abdominal pain, fever)	epsilon1	NT	stx2	-	N	this study
O76:H-	08E035	2008	human	Japan	asymptomatic carrier	gamma1	NT	stx1	-	Y	this study
O165:H-	08E132	2008	human	Japan	symptomatic (bloody stool, abdominal pain, vomitting, fever)	epsilon1	NT	stx1&2	-	N	this study
O165	osen07-074	2007	Bovid	Japan	food	epsilon1	NT	stx2	-	N	this study
OUT	CB10113	2004	cat	Brazil	domestic (asymptomatic carrier)	ypsilon	pheU	-	-	Y‡	Morato *et al.*, 2009ƒ
ONT	CB9637	2003	human	Germany	symptomatic (diarrhea)	rho	pheU	-	-	N	this study
O180	CB9776	2003	human	Germany	symptomatic (diarrhea)	rho	pheU	-	-	Y	this study
O65	CB9786	2003	human	Germany	symptomatic (diarrhea)	alpha8	pheU	-	+	Y‡	this study
O168	CB9791	2003	human	Germany	symptomatic (diarrhea)	alpha8	pheU	-	+	Y‡	this study
O180	DG172/5	1990	sheep	Germany	asymptomatic carrier	rho	pheU	-	-	N	this study
NT:H19	0471-1	1989	human	Brazil	symptomatic (diarrhea)	rho	pheU	-	-	Y	Ooka *et al.*, 2008∫
NT:HNM	4051-6	1989	human	Brazil	symptomatic (diarrhea)	omicron	pheU	-	+	Y‡	Ooka *et al.*, 2008∫
NI	A09/332.1	2008.11	deer	Belgium	*Capreolus capreolus* (asymmptomatic)	epsilon2	selC	-	-	Y	Bardiau *et al.*, 2010¶
O115:HNM	HIPH08472	2008.8	human	Japan	symptomatic (diarrhea)	N2	pheU	stx2f	+	Y‡	this study
OUT:H-	E2675	2007	bird	Japan	feces swab, Corvus spp.	N1.2	pheU	stx2f	+	Y‡	this study
O156:H25	RIMD05091872	2003	human	Japan	asymptomatic carrier	zeta	pheV	stx1	-	Y	this study
O55:H6	F76193	NI	human	SSI	symptomatic (diarrhea)	alpha2	selC	-	-	Y	Iida *et al.*, 2001§
O153:HNM	HIPH07217	2007.8	human	Japan	symptomatic (diarrhea, fever)	beta1	NT	stx2f	+	Y	this study
O63:H6	HIPH07137	2007.8	human	Japan	symptomatic (diarrhea, abdominal pain)	alpha2	selC	stx2f	-	Y	this study
O145:H34	HIPH08592	2008.10	human	Japan	symptomatic (diarrhea, fever)	iota1	selC	stx2f	+	Y	this study
O128:HNM	EC2175	2002.7.28	human	Japan	symptomatic (one year old, diarrhea, bloody mucus stool, vomitting)	beta1	NT	stx2f	+	Y	this study
O63:H6	EC2689	2006.9.16	human	Japan	symptomatic (four years old, fever, cough, soft stool)	alpha2	selC	stx2f	+	N	this study
O145:H34	E2473	2006.8	human	Japan	symptomatic (diarrhea, bloody stool, abdominal pain, fever)	iota1	selC	stx2f	+	N	this study

Serotype†	strain name	year of isolate	origin	countries	symptoms or notes	intimin subtypes	LEE integration sites (tRNA gene)	presence or absence		MLST analysis	References
								stx1&2&2f	cdtB		
O63:H6	A32	2003.8	human	Japan	symptomatic (diarrhea, abdominal pain)	alpha2	selC	stx2f	-	N	this study

NI: no infomation, NT: not typed
†: Determined by the serotyping system for *E. coli*.
‡: *E. albertii* strains (confirmed by MLS analysis).
ƒ: Morato *et al.* (2009) Domestic cats constitute a natural reservoir of human enteropathogenic *Escherichia coli* Types. Zoonoses Public Health. 56: 229-237.
∫: Ooka T *et al.* (2007) Characterization of tccP2 carried by atypical enteropathogenic *Escherichia coli*. FEMS Microbiol Lett. 271: 126-135.
¶: Bardiau M *et al.* (2010) Enteropathogenic (EPEC), enterohaemorragic (EHEC) and verotoxigenic (VTEC) *Escherichia coli* in wild cervids. J Appl Microbiol. 109: 2214-2222.
§: Iida K *et al.* (2001) Type 1 fimbtiation and its phase switching in diarreagenic *Escherichia coli* strains. Clin Diagn Lab Immunol. 8: 489–495.

Technical Appendix Table 2. PCR primers for detection and sequencing of the *stx* and *cdt* genes

target gene	primer name	sequence (5'-3')	PCR conditions (30 cycles)	Size of amplicon (bp)	References
5' half of *eae*	cesT-F9	TCAGGGAATAACATTAGAAA	92⌡C, 60 s / 55⌡C, 60 s / 72⌡C, 2 min	around 1.3 kb	Lacher *et al.*, 2006ƒ
	eae-F1	ACTCCGATTCCTCTGGTGAC			
3' half of *eae*	eae-R3	TCTTGTGCGCTTTGGCTT	92⌡C, 60 s / 52⌡C, 60 s / 72⌡C, 2 min	around 1.6 kb	Lacher *et al.*, 2006ƒ
	escD-R1	GTATCAACATCTCCCGCCCA			
inside of *eae*	1669-1688§	CAGGTTGGGGTAACGGACTT			this study
stx1	stx1-F	GTCATTCGCTCTGCAATAGGTAC	94⌡C, 30 s / 64⌡C, 30 s / 72⌡C, 90 s	151	Ooka *et al.*, 2009ʃ
	stx1-R	GCCGTAGATTATTAAACCGCCCT			
stx2, *stx2c*, *stx2d*, *stx2e*	stx2-F	CCATGACAACGGACAGCAGTT	94⌡C, 30 s / 64⌡C, 30 s / 72⌡C, 90 s	181	Ooka *et al.*, 2009ʃ
	stx2-R	CTGCTGTGACAGTGACAAAACG			
stx2f	128-1	AGATTGGGCGTCATTCACTGGTTG	94⌡C, 30 s / 57⌡C, 60 s / 72⌡C, 60 s	428	Schmidt *et al.*, 2000¶
	128-2	TACTTTAATGGCCGCCCTGTCTCC			
cdtB type I and IV	CDT-s2	GAAAATAAATGGAACACACATGTCCG	94⌡C, 60 s / 55⌡C, 60 s / 72⌡C, 60 s	466	Toth *et al.*, 2003†
	CDT-as2	AAATCTCCTGCAATCATCCAGTTA			
cdtB type II, III, V	CDT-s1	GAAAGTAAATGGAATATAAATGTCCG	94⌡C, 60 s / 55⌡C, 60 s / 72⌡C, 60 s	466	Toth *et al.*, 2003†
	CDT-as1	AAATCACCAAGAATCATCCAGTTA			

§: This primer is used only for sequencing of the *eae* gene.
ƒ: Lacher DW *et al.* (2006) Allelic subtyping of the intimin locus (*eae*) of pathogenic *Escherichia coli* by fluorescent RFLP. FEMS Microbiol Lett. 261:80-87.
ʃ: Ooka T *et al.* (2009) Development of a multiplex PCR-based rapid typing method for enterohemorrhagic *Escherichia coli* O157 strains. J Clin Microbiol. 47:2888-2894.
¶: Schmidt H *et al.* (2000) A new Shiga toxin 2 variant (Stx2f) from *Escherichia coli* isolated from pigeons. Appl Environ Microbiol. 66:1205-1208.
†: Toth I *et al.* (2003) Production of cytolethal distending toxins by pathogenic Escherichia coli strains isolated from human and animal sources: establishment of the existence of a new cdt variant (Type IV). J Clin Microbiol. 41:4285-4291.

Technical Appendix Table 3. Reference sequences of the *eae* genes

subtype	species of origin	Serotype	Strain name	accession No.
α1 (alpha1)	*Escherichia coli*	O127:H6	E2348/69	AF022236
α2 (alpha2)	*Escherichia coli*	O125:H6	C712-65	DQ523600
α8 (alpha8)	*Escherichia albertii*	-	I2005002880 36	FJ609835
β1 (beta1)	*Escherichia coli*	O26:H-	413/89-1	AJ277443
β2 (beta2)	*Escherichia coli*	O119:H6	0659-79	DQ523605
β3 (beta3)	*Escherichia coli*	-	AEEC-H03/34136b	AJ876654
ε1 (epsilon1)	*Escherichia coli*	O103:H2	MT#80	DQ523606
ε2 (epsilon2)	*Escherichia coli*	O116:[H9]	98B3	DQ523614
ε3 (epsilon3)	*Escherichia coli*	-	AEEC-H03/31923a	AJ876649
ε4 (epsilon4)	*Escherichia coli*	-	AEEC-H03/37159a	AJ876651
η (eta)	*Escherichia coli*	O142:[H21]	012-050982	DQ523604
η2 (eta2)	*Escherichia coli*	-	AEEC-H03/53199a	AJ876652
γ1 (gamma1)	*Escherichia coli*	O157:H7	Sakai	BAB37982.1
γ2 (gamma2)	*Escherichia coli*	O111:H-	95NR1	AF025311
ι1 (iota1)	*Escherichia coli*	O55:[H34/47]	1252-59	DQ523601
ι2 (iota2)	*Shigella boydii*	13	C-425	AY696842
κ (kappa)	*Escherichia coli*	O49:[H10]	64B4	DQ523611
λ (lambda)	*Escherichia coli*	O33:[H34]	57A1	DQ523609
μ (mu)	*Escherichia coli*	O55:[H51]	MA551/1	DQ523607
ν (nu)	*Escherichia albertii*	-	106A5	DQ523615
ο (omicron)	*Escherichia albertii*	-	19982	AY696838
π (pi)	*Escherichia coli*	-	AEEC-191.2	AJ705052
ρ (rho)	*Escherichia coli*	93I4	93I4	DQ523613
σ (sigma)	*Escherichia coli*	O86:K61:H-	EPEC-EC74699	AJ781125
τ (tau)	*Shigella boydii*	7	K-1	AY696839
θ (theta)	*Escherichia coli*	O111:H8	CL-37	AF449418
ξ (xi)	*Escherichia coli*	O5:[H2]	60A3	DQ523610
υ (ypsilon)	*Escherichia coli*	ONT	CB10113	AM116755.1
ζ (zeta)	*Escherichia coli*	-	921-B4	AF449417
ζ3 (zeta3)	*Escherichia coli*	O85:H31	FV10126	FM872423
C. rodentium	*Citrobacter rodentium*	-	DBS100	AF311901

Technical Appendix Table 4. Shiga toxin production by the two *stx2f*-positive *E. albertii* strains

Strain name	Species	prevalence of *stx* genes	VTEC-RPLA†				References
			Stx1		Stx2		
			MMC+	MMC-	MMC+	MMC-	
HIPH08472	*E. albertii*	*stx2f*	-	-	+ (64)	+ (2)	this study
E2675	*E. albertii*	*stx2f*	-	-	+ (8)	-	this study
LMG20976 (type strain)	*E. albertii*	-	-	-	-	-	this study
CB9786	*E. albertii*	-	-	-	-	-	this study
O128:HNM EC1463	*E. coli*	*stx2f*	-	-	+ (> 128)	+ (8)	Isobe *et al.*, 2004∫
O157:H7 Sakai	*E. coli*	*stx1*, *stx2*	+ (> 128)	+ (>128)	+ (> 128)	+ (32)	Hayashi *et al.*, 2001§

†: The maximum dilution of culture supernatant that exhibited agglutination is shown in parentheses.
∫: Isobe J *et al.* (2004) Isolation of *Escherichia coli* O128:HNM harboring *stx2f* gene from diarrhea patients Kansenshogaku Zasshi. 78: 1000-1005.
§: Hayashi T *et al.* (2001) Complete genome sequence of enterohemorrhagic *Escherichia coli* O157:H7 and genomic comparison with a laboratory strain K-12. DNA Res. 8: 11-22.

Technical Appendix Table 5. PCR primers for determination of the LEE integration sites†

primer name	sequence (5'-3')	target locus	location of the primers on the sequenced *E. coli* strains	Expected amplicon size (bp)	References
escR-R	ACTGGCGATACCATCATCATAC	-	escR gene on the LEE core region	-	this study
pheV-Ro1	CAGGTATGTACCTTCACCGTTGG	*pheV*	26,337 bp downstream of the *pheV* 3'-end of K-12 strain MG1655	around 30 kb	this study
pheV-glcB	ACAATGAGTCAAACCATAACCCA	*pheV*	13,367 bp downstream of the *pheV* 3' end of K-12 strain MG1655	around 17 kb	this study
selC-Ro1	CACGGCGGCAATCAGAACGTTC	*selC*	1,984 bp downstream of the *selC* 3'-end of O103:H2 strain 12009	around 5 kb	this study
433-f	ACGCGGGATTGGTTTTGGTCAG	*pheU*	14,687 bp downstream of the *pheU* 3'-end of O157:H7 strain Sakai	around 18 kb	Ohnishi *et al.*, 2002§

†: PCR cycle; 2 min at 96°C, followed by 30 cycles of 20 s at 96°C and 16 min at 69°C.

§: Ohnishi M et al. (2002) Genomic diversity of enterohemorrhagic Escherichia coli O157 revealed by whole genome PCR scanning. Proc Natl Acad Sci USA. 99:17043-17048.
